# Gut permeability and osteoarthritis, towards a mechanistic understanding of the pathogenesis: a systematic review

**DOI:** 10.1080/07853890.2021.2014557

**Published:** 2021-12-21

**Authors:** Giorgio Guido, Guido Ausenda, Veronica Iascone, Emanuele Chisari

**Affiliations:** a Department of General Surgery and Medical-Surgical Specialties, University of Catania, Catania, Italy; b Faculty of Medicine, University of Milan, Milan, Italy; c Experimental, Diagnostic and Specialty Medicine, University of Bologna, Bologna, Italy; d Rothman Orthopaedic Institute at Thomas Jefferson University, Washington Township, NJ, USA

**Keywords:** Osteoarthritis, tight junctions, gut microbiota, microbiome, intestinal permeability, joint inflammation

## Abstract

Osteoarthritis (OA) is the most common condition affecting human joints. Along with mechanical and genetic factors, low-grade inflammation is increasingly supported as a causal factor in the development of OA. Gut microbiota and intestinal permeability, *via* the disruption of tight junction competency, are proposed to explain a gut-joint axis through the interaction with the host immune system. Since previous studies and methods have underestimated the role of the gut-joint axis in OA and have only focussed on the characterisation of microbiota phenotypes, this systematic review aims to appraise the current evidence concerning the influence of gut permeability in the pathogenesis of OA. We propose that the tight junction disruption may be due to an increase in zonulin activity as already demonstrated for many other chronic inflammatory disorders. After years of unreliable quantification, one study optimised the methodology, showing a positive validated correlation between plasma lipopolysaccharide (LPS), obesity, joint inflammation, and OA severity. Chemokines show a prominent role in pain development. Our systematic review confirms preliminary evidence supporting a gut-joint axis in OA pathogenesis and progression. Being modifiable by several factors, the gut microbiota is a promising target for treatment. We propose a pathogenetic model in which dysbiosis is correlated to the bipartite graph of tight junctions and bacterially-produced products, aiming to direct future studies in the search of other bacterial products and tight junction disassembly regulators.

**KEY MESSAGES**
- Previous studies and methods have underestimated the impact of the gut-joint axis in osteoarthritis and have focussed on the characterisation of microbiota phenotypes rather than clear molecular mediators of disease.
- Gut dysbiosis is related to higher levels of bacterial toxins that elicit cartilage and synovium inflammatory pathways.
- Future research may benefit from focussing on both tight junctions and bacterially-produced products.

## Introduction

Osteoarthritis (OA) is the most common condition affecting human joints and the 15^th^ highest cause of years lived with disability worldwide [1]. Though it may develop in any joint, it predominantly affects diarthrodial joints such as the hip and, following disease progression, it ultimately leads to joint failure [2]. As our understanding of the pathophysiology continues to evolve, we have come to realise that OA is a complex disease with multifactorial aetiology, where an interplay between host and environmental factors instigate the disease [3,4]. However, it is not known why the progression of the disease is highly variable in the individuals who develop this condition. Despite traditionally considered “non-inflammatory” arthritis, low-grade inflammation seems to have a causal role in OA, and new studies suggest that it may be triggered by the complex interplay between the gastrointestinal microbiome [5], its products, and the immune system.

The gut microbiome comprises more than 3 million genes, and each person features a unique microbiome composition [6]. Taxonomic studies report that *Firmicutes* (including *Lactobacillus, Bacillus, Clostridium, Enterococcus,* and *Ruminicoccus* genera) and *Bacteroidetes* (including *Sphingobacterium*, *Bacteroides, Alistipes, Prevotella* genera) are the main phyla, representative of 90% of the total gut microbiome [7]. Many studies suggest that several intestinal [8] and extra-intestinal diseases [9] are associated with specific bacterial motifs and dysbiosis, that is a reduction in microbial diversity and functional imbalance in microbial communities. Notwithstanding the absence of proof for a causal relationship between dysbiosis and pathophysiology, for several diseases, a role in clinical severity is demonstrated [10]. Gut permeability is an emerging area of microbiome research since has been shown as pivotal in non-gut disorders [11], and diseases traditionally considered on a purely autoimmune basis. In particular, evidence for a significant role of the microbiota in the modulation of tolerogenic mechanisms has been reported [12,13].

Traditionally, low-vascularized tissues like articular cartilage were considered the core of OA pathogenesis. Thus, the joint environment seemed to be influenced by systemic factors either indirectly through the subchondral bone or in the neo-angiogenesis phase of OA pathogenesis [14] when a multitude of blood molecules, including bacteria and bacterial products, have a gate from the blood to the cartilage [15–17]. However, on a more accurate investigation, findings suggest that the synovium is part of a dynamic interplay inside the joint in OA development. Altogether, these findings cannot be dismissed as pathological [18], bacteria and bacterial products could affect the epigenetic landscape of chondrocytes [19] and prime the innate immune response in the joint *via* Toll-like receptors signalling [5]. In addition, a recent study [20] suggests that a microbiome exists inside the OA knee and hip joints. The overall ecological interactions among these microbial patterns are still mainly unknown.

How the gut influences OA pathogenesis is not clear. Limited attention has been paid to the role of intestinal permeability as a mediator of the effects of the microbiome on the joint. Previous studies and methods have underestimated the impact of microbiome products and intestinal permeability in the pathogenesis of OA [21]. Given the lack of mechanistic insight and the absence of a clear definition of the molecular actors involved in the pathogenesis of OA, in this review, we aim to appraise the direct and indirect effects of gut dysbiosis on the affected joint, focussing on clear molecular mediators emerging from related literature, to help direct future studies.

## Materials and methods

### Study search strategy

**Figure 1.**
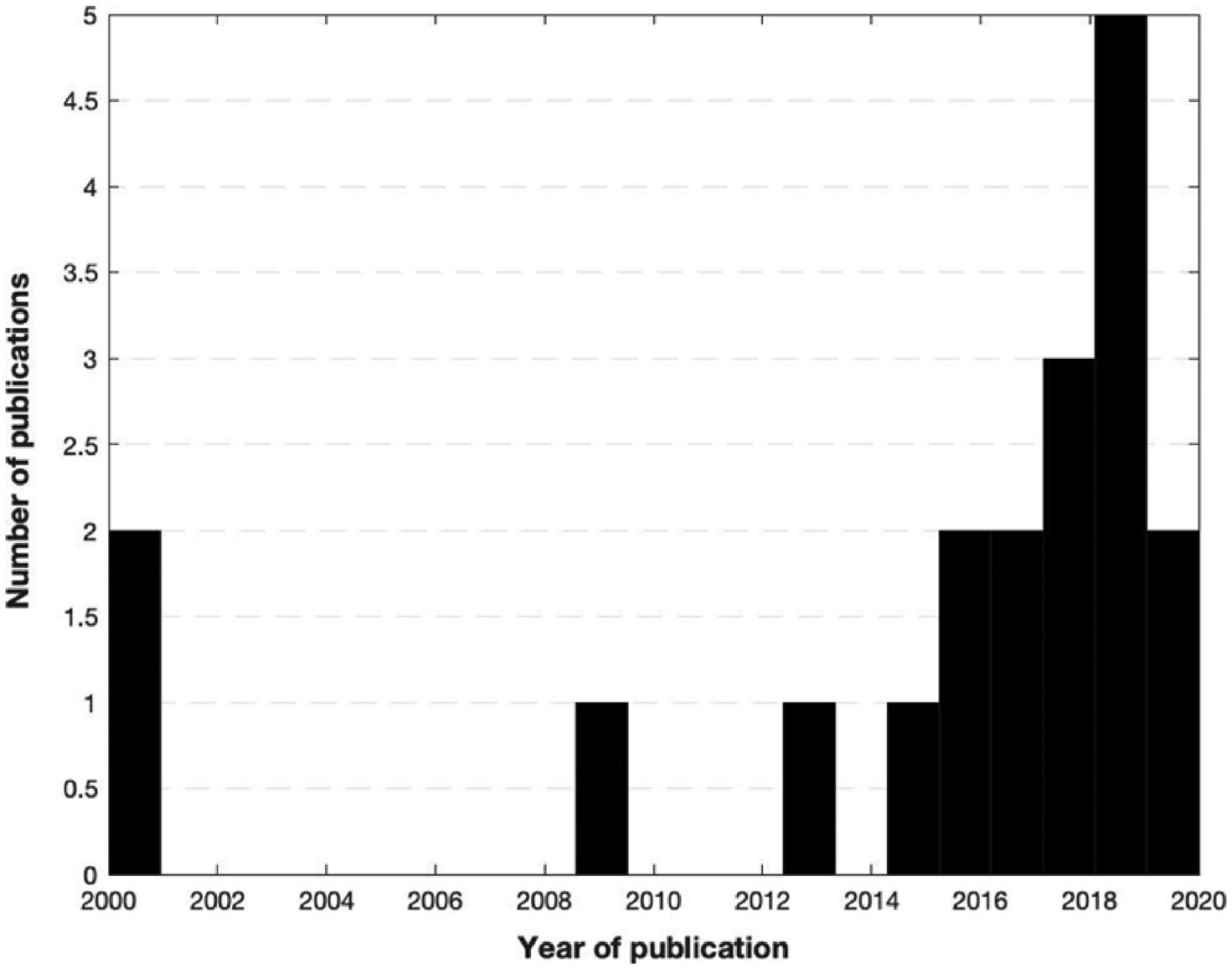
A number of publications about microbiome and osteoarthritis selected per year.

**Figure 2.**
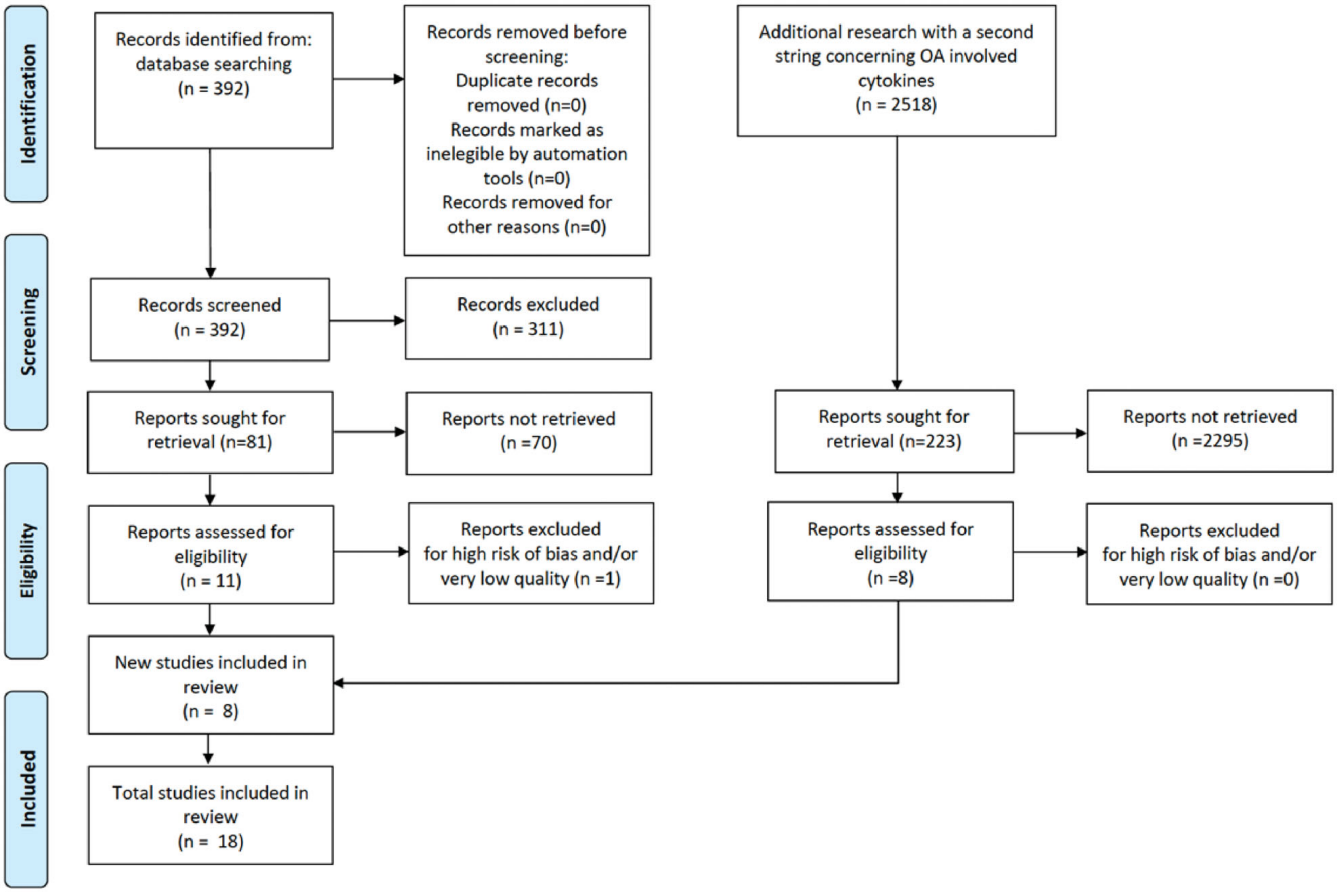
PRISMA flow diagram of study selection.

This systematic review was conducted according to the 2021 guidelines of the Preferred Reporting Items for Systematic Reviews and Meta-Analyses (PRISMA) (Figure 2) [22]. A comprehensive search was performed using three electronic medical databases (PubMed, EMBASE, and Cochrane Library) by two independent authors (GA and VI) from January 2020 to April 2020. To achieve maximum sensitivity we combined the terms “gut microbio* OR gut OR streptococcus OR lactobacillus OR bifidobacterium OR clostridium OR microbiome” and typical anatomical landmarks of disease “hip OR knee” with some terms related to osteoarthritis and inflammation (arthritis, osteoarthritis, inflammation, synovial, synovitis, cytokines, chemokines, molecular mediators) as either keywords or Medical Subject Heading (MeSH) terms. To elucidate the growing interest in the review topic in the medical literature, we graphically summarised the number of articles per year (Figure 1). The research was then repeated in February 2021 but did not retrieve new results.

**Figure 3.**
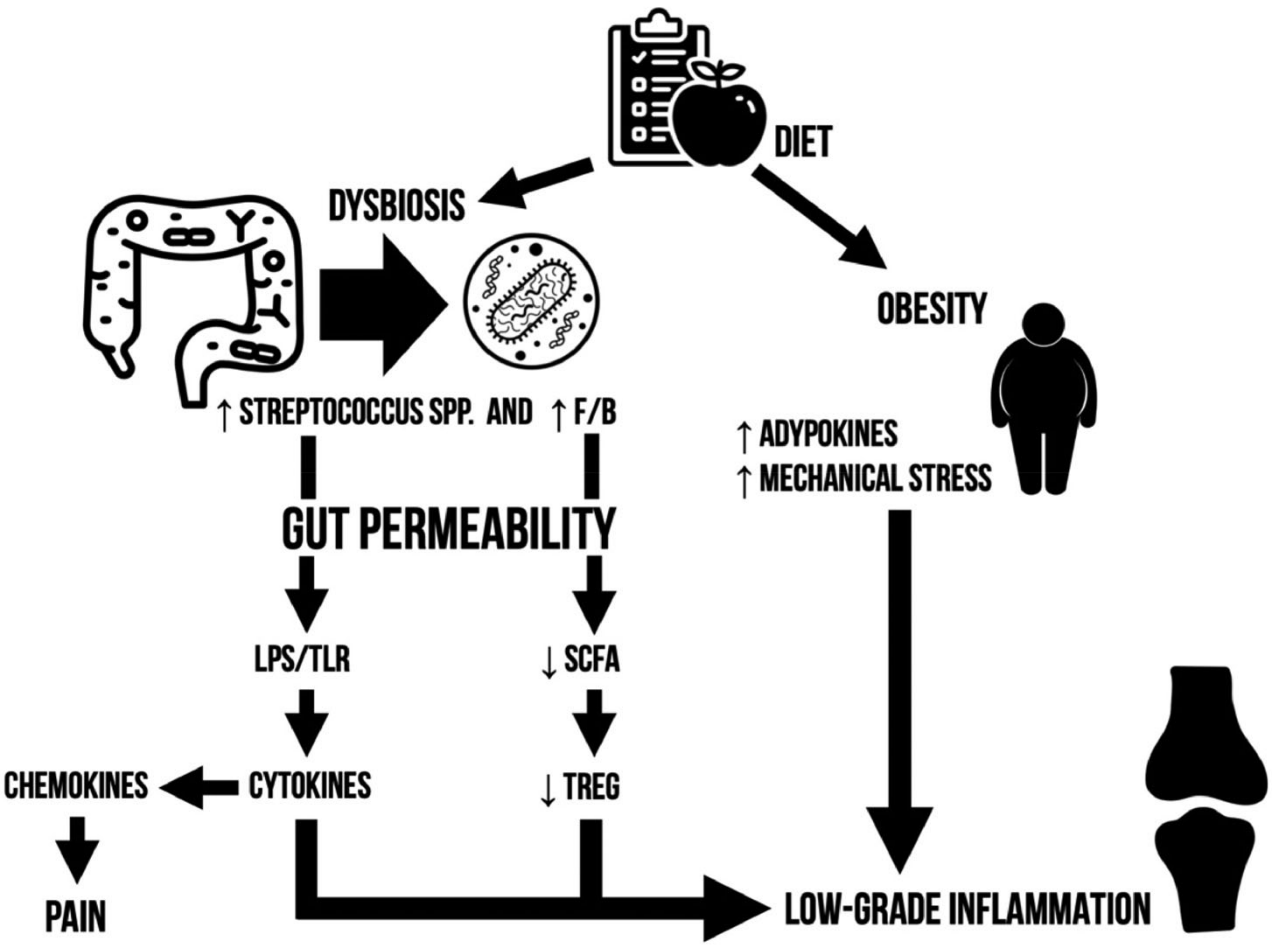
Graphical summary of microbiome-mediated osteoarthritis pathogenesis.

References of all included articles, previous reviews, and Google Scholar top results were reviewed to identify further relevant studies. Moreover, intending to avoid overlap with other ongoing systematic reviews, we searched PROSPERO for any similar work.

### Selection criteria

Selected studies included those investigating the influence of microbiome composition on osteoarthritis, the role of diet and dietary interventions on the inflammatory state, and the molecular mediators involved. Both clinical and preclinical studies published in English in peer-reviewed journals were screened. We excluded studies with missing or not accessible data. We excluded studies for which a full-text article was not available, as well as not well-reported studies. We excluded duplicates and studies with a poor or unclear methodology. Finally, we excluded reviews, case reports, conference presentations, and articles only containing opinions. Three authors (GA, GG and VI) searched and evaluated the articles independently. An experienced researcher (EC) resolved cases of doubt. All abstracts were read and, according to inclusion and exclusion criteria, relevant articles were selected. A month later, the rereading of the same studies ensured agreement among the investigators. One investigator (GA) extracted data from full-texts into Excel to analyse each study and data were double-checked by the other two investigators (GG, VI).

### Data extraction and criteria appraisal

Data were extracted from article texts, tables, and figures using the Population, Intervention, Comparison, Outcome framework [23]. Title, year of publication, study design, number and characteristics of the subjects involved were considered together with study outcomes and conclusions. Three investigators (GA, GG, and VI) independently reviewed each article. Discrepancies were resolved by discussion.

### Risk of bias assessment

The risk of bias of 4 non-randomized clinical studies was assessed according to the ROBINS risk of bias tool [24]. This tool used “low,” “moderate,” and “high” to describe the risk of bias. The assessment was performed by two authors (GA and VI) independently, with an inter-rater agreement of 90%. Any discrepancy was solved by consensus. 3 studies had a moderate risk of bias, and 2 studies had a low risk of bias. One article was excluded because of a serious risk of bias. The risk of bias assessment for pre-clinical studies was instead performed using the SYRCLE’s tool [25]. A score ranging from 0 (lowest) to 10 (highest) was used. Two investigators evaluated the studies independently (GA and VI) with 95% agreement. The risk of bias for the included studies was low to moderate (mean SYRCLE score 4.4, range 3–6) and no studies were excluded following this assessment.

### Certainty assessment

The certainty of clinical studies was assessed using GRADE [26]. According to this method, each study was classified as “very low”, “low”, “moderate” or “high”. With the exception of one study that featured low confidence of evidence, all the studies ranked either “high” (3 studies) or “moderate” (2 studies).

The Collaborative Approach to Meta-Analysis and Review of Animal Data from Experimental Studies (CAMARADES) checklist [27,28] was used to assess the certainty of the pre-clinical studies (*n* = 13). The assessment was performed independently by two authors (GA, VI) with 94% agreement. Each study was assessed and scored on a scale from 0 (lowest) to 10 (highest) points. The overall certainty was moderate among the included studies (mean CAMARADES score 4.3, range 3–6).

## Results

### Study characteristics

We included results from 18 studies. 13 were performed on animal models [18,29–40] and 5 were human observational studies [5,21,41–43]. The models investigated were adult mice [21,29,30,32,33,36,38] and adult rats [18,34,37]. In the clinical studies included, the population was aged between 50 and 65 years.

Most of the considered studies analysed the relationship between OA and the microbial populations’ variations in the gastrointestinal tract or investigated cytokines with a mostly documented role in OA pathogenesis [30–33,39–43]. Metabolic alterations of the microbiome and their correlation to gut permeability were investigated through the serum concentration of LPS [18,21,34,35,38]. Diet and obesity were analysed concomitantly to the microbiome in mice [18,34,35]. Some studies analysed the role in OA severity of physical exercise [34] and other dietary aspects: the administration of nutritional supplementation [37] and/or prebiotics in rodents [34–37] and humans.

### Dysbiosis-related gut permeability

When gut dysbiosis was induced by a high-fat diet (HFD), high-sugar diet, faecal transplant or sex steroid deficiency, higher levels of bacterial toxins and gut bacterial translocation to the blood were retrieved [34,35]. One study transplanted the faecal microbiota of patients with metabolic syndrome in mice, and assessed gut permeability, detecting lower mRNA levels of tight junction proteins (TJs), zonulin-1 (ZO-1) and occludin, and higher LPS plasma levels. Bacterial translocation *via* the intestinal endothelium was also confirmed using FISH methodology [29].

It was hypothesised that oligofructose could prevent gut dysbiosis and potentially reduce the OA of the obesity retrieved in previous animal studies [35]. While oligofructose supplementation in mice did not prevent the onset of obesity, it was shown that the gut had improved epithelial function, and bacterial products levels, such as LPS serum levels, were found to be lower [35]. This was then found to be associated with lower cartilage degeneration in the interventional group [35]. These effects seem to be mediated by the ability of oligofructose to partially alter the gut microbiome relative abundance avoiding the total loss of *Bifidobacterium,* which is commonly observed in mice obesity models [18,34,35]. Similar results were replicated by other pre- and probiotics [34,36,37]. Moreover, oligofructose administration was found to upregulate Cdx2, a transcription factor regulating cell adhesion, and increase Grp (a stimulator of epithelial proliferation in the intestine) and Aqp4 (involved in water reabsorption), all related to the maintenance of normal intestinal permeability [35].

### Bacterial products contribute to low grade inflammation in the joint

Germ-free mice with destabilised medial meniscus showed a reduction in lipopolysaccharide-binding protein (LBP) but not LPS due to difficulty in blood LPS quantification [38]. When optimised methodology to assess LPS was performed, a positive association of synovial LPS with inflammation and disease severity was reported [21]. Articular chondrocytes mount an LPS-induced stress response *via* Toll-like receptors (TLRs) (especially TLR4) and secrete matrix metalloproteinases as well as innate immune mediators [46]. Fibroblast-like synoviocytes from OA patients upregulate their expression of the inflammasome receptors NLRP1/3 upon *in vitro* stimulation with LPS [47].

Firmicutes/Bacteroides (F/B) phyla ratio increase in gut dysbiosis models has shown to be mostly lead by a decrease in *Bacteroidetes* phylum population, whereas the absolute Firmicutes abundance resulted almost unchanged, but showed significant qualitative changes, particularly a decrease in *Lactobacillus spp.* and an increase in *Clostridiales* order [18]. In addition, *Streptococcus spp.,* have shown to represent 20% of total *Firmicutes* in OA patients of a large population cohort study [5] and resulted positively correlated with higher OA pain scores and lower functionality [5]. Bacterial *toxins* may therefore connect the increased gut permeability to low-grade inflammation in OA.

### Molecular mediators of inflammation and joint pain

Obese mice displayed a 5-fold increase in the number of infiltrating macrophages [35] and increased analytes: CXC-family chemokines (KC, MIG), adiponectin, leptin, IL-1Ra [44], IP-10, MCP-1, MIP-2 and MIP-1alfa [18]. A role for IL-18 [39] and IL-12 [40] was identified. From several studies balance between pro- and anti-inflammatory mediators emerges as key [36,37,39,40].

The innate immune pro-inflammatory cytokine IL-1beta was shown to up-regulate aquaporins AQP1 and AQP3 in an OA model. These molecules explain the joint swelling in early OA, and the subsequent loss of proteoglycans and chondrocytes apoptosis that accelerate the progression of the disease [30].

Among all the molecular mediators, chemokines have been shown to have a prominent role in the establishment of pain. While many chemokines show a mixed role in pain, chemoattraction and disease progression [31,41–43], the CCL2/CCR2 axis plays a predominant role in the development of pain, as confirmed by many studies [32,33,45].

## Discussion

### Gut permeability as a foundational element of the gut-joint axis

Gut microbiome changes are acute and precede obesity in mouse models. Indeed, obesity is associated with impairment of gut mucosa and microbiome translocation [46]. Interestingly, partaking in exercise, together with weight loss, is among the strong recommendations based on sound evidence for OA control [47]. Both happen to be associated with reduced pain and disease progression [48,49], and along with nutraceutical use [36,50], these interventions have been shown effective in reverting the acute microbiome compositional changes associated with OA.

However, when gut dysbiosis occurs either for chronic disease (e.g. inflammatory bowel disease, immunosuppression), chronic antibiotic treatment, or lifestyle modification (e.g. obesity and metabolic disease), gut permeability is significantly affected [51–56]. HFD modulates tight junctions, their expression and distribution, directly through dietary fats or indirectly *via* the increase in cytokines release [57]. HFD not only reduces TJ molecules but also depletes eosinophils associated with correct gut permeability [58], reduces mucus and antimicrobial peptides production [59]. Moreover, microbes are spatially redistributed in the intestine, mainly occupying intervillous/cryptal spaces [60].

If such changes do occur, progressively, innate immune receptors in the gut get activated by microbial products and stimulate pro-inflammatory mediators production. Pro-inflammatory cytokines, in turn, dysregulate TJs formation creating a vicious cycle. TNFα, for instance, is known to be involved in occludin internalisation, while IFNγ reduces both ZO-1 and occludin expression. Myosin light chain kinase (MLCK) seems to be important in the cytokine-mediated regulation of TJ complexes [61]. As a general view, TJ dysregulation may be induced by cytokines, by immune cells, by NSAIDs, or alcohol chronic use, as well as by pathogens in the context of a dysbiotic microbiome [62].

It is indeed a well-known fact that many enteric pathogens produce toxins that affect the host’s tight junctions [63]. In particular, given that TJ competency was found to be affected *via* a decrease in ZO-1 and occludin mRNA levels in the analysed studies, we propose that this tight junction disruption may be in part due to an increase in zonulin activity, already demonstrated for many chronic inflammatory and autoimmune disorders [64]. Zonulin is a physiologic regulator of intestinal permeability but is known to be pathologically triggered by gliadin and by bacteria in the gut. Zonulin release seems to be dependent on MyD-88 [65]. Inappropriate zonulin activity has been shown for diseases as varied as coeliac disease, ankylosing spondylitis, anxiety and depression (in a dysbiosis-mediated manner) and multiple sclerosis, in which zonulin may explain increased permeability to both the gut and the blood-brain barrier [66–68]. Similarly, we propose that zonulin may be involved in the gut-joint axis of osteoarthritis. TJ disruption provides a framework to unify several inflammatory diseases under the common denominator of permeability, then interact with the patient genetic background [69].

### Dysbiosis perturbates the delicate balance between pro- and anti-inflammatory mediators

Our main findings support an increase in *Firmicutes/Bacteroidetes* (F/B) phyla ratio [18,34,35]. Given the decrease in Bacteroidetes and their ability to produce high levels of *short-chain fatty acids*, which regulate the differentiation of *Treg* cells [70], and the role of butyrate, which has been shown to be able to regulate zonulin [71,72], this particular enteric phenotype may further influence intestinal permeability, summing to the above mechanisms.

Another key feature of the OA dysbiotic enterotype is *Streptococcus spp*. the abundance which seems to be critical to prime both local and systemic inflammation through LPS-induced macrophage activation [5] *via* the *NFkB* or *MAPK* pathways [73], or by forming complexes with LBP and CD14 [21]. Mechanistic evidence was reported with increased CD14 levels in the synovium of OA patients compared to healthy controls [74]. Macrophages can be detected not only in the synovium but also in the synovial fluid and in the peripheral blood [75]. CX3CR1-expressing macrophages have been found to form a highly dynamic barrier (expressing epithelial genes and tight junctions) in the lining layer of the synovium, adjacent to fibroblasts [76]. This barrier undergoes remodelling in arthritis models, thus single-cell studies of macrophage populations are warranted in OA-specific models. Moreover, the *M1/M2 macrophages ratio* determines the severity of the disease. While the former induces chondrocyte apoptosis and decreases the synthesis of extracellular matrix components, the latter, through *TGFβ* expression, stimulates chondrogenesis and *collagen II*/p*roteoglycan* formation [77].

Thus, an increase in F/B ratio and *Streptococcus spp*. prevalence are both able to ultimately dysregulate the delicate balance between pro-inflammatory markers (*IL-1beta, IL-6, TNFα, IL-12, IL-18*) at the expense of anti-inflammatory molecules (*IL-4, IL-10, TGFβ*) [37]. Moreover, the role of the key pro-inflammatory cytokine IL-1beta in the local deterioration of the joint *via* aquaporins [78] may suggest a further probable mechanism, mediated by other inflammatory mediators.

It is important to notice that studies regarding the microbiota in various diseases have shown a poor concordance of findings, mainly due to the dynamic nature of bacterial presence in the gut [79]. More than 50% of the characterised bacterial species in a recent study [80] were never described before, and this raises concerns about the possibility of spurious associations, limiting our capacity to extrapolate solid causal relationships. Therefore, we suggest focussing on small molecules, such as bacterially produced products and immune mediators, rather than singular bacterial species in order to gain useful mechanistic insight.

### Endotoxemia

An evidence-based gut-joint axis clearly stems from many of the studies. Both inflammatory mediators and bacterial toxins are translocated into the circulation [81] which raises concerns in patients that have joint prosthetic implants or artificial valves at high risk of infection [82,83]. The increased leucocytes and macrophage presence [35,84] may possibly let them act as a “Trojan Horse” [85–87] from the gut to the joint. The articular cartilage does not receive nutrition from blood vessels, instead, support is provided on one side by the synovial fluid and on the other by the subchondral bone marrow. Systemic bacterial toxins, inflammatory mediators and blood microbiota [88] can thus influence the joint in two ways: *via* large cracks of the articular cartilage [89] or *via* vascular channels arising from neoangiogenesis occurring at the osteochondral junction [90]. Moreover, given the aforementioned potent effects of HFD, meals might trigger pulsatile increases in LPS or other bacterial products in the blood, contributing to chronic low-grade inflammation. Experimental support or confutation to this idea is warranted. The circadian response to endotoxemia should also be assessed in the context of OA given the interesting outcome of some preliminary animal studies [91]. This may inform lifestyle modifications in these patients. Another level of complexity is added by the finding that LPS elicits distinct immune responses based on the bacterial species of origin [92]. An analysis of LPS subtypes, rather than the mere quantification of them, may provide further insight.

### Pain symptomatology and chemokines

Studies show that the microbiome also relates to pain-associated symptoms and molecules [93]. The main actors are cartilage degradation products (following mechanical and inflammatory stress on the joint) and other damage-associated molecular patterns through direct neuronal activation of dorsal root ganglia or by indirect neuro-immune signalling acting on immune cells receptors that in turn stimulate neurons amplifying the mechanism. Even if cytokines and LPS partially explain the onset of pain, *chemokines* are reported as critical players of chronic pain [84]. Consistent with this interpretation, in our results, chemokines were regularly increased among all the considered analytes [35].

## Conclusions

### Future research directions

In order to be able to translate these findings, research priorities need to be identified. The pathogenetic model herein discussed correlates dysbiosis to the bipartite graph of tight junctions and bacterially produced products (Figure 3). Putting the accent on these two key features we aim to direct future studies in the search of bacterial toxins/products other than LPS and tight junction complexes disassembly regulators. However, we recommend caution since quantification assays commonly encounter reliability and reproducibility issues.

### Future therapeutical perspectives

Current treatments for OA alleviate pain but do not target the pathogenesis of the disease [94]. Being modifiable by several factors (dietary intervention, faecal transplant, and future microbiome-targeted therapeutics), the gut microbiome is a promising target. The first line of action is exercise, diet control and if the proposed role for zonulin is confirmed, gluten-poor food. Targets such as zonulin and aquaporins as well as the pyroptosis pathways (gasdermin blockers), might be further investigated pharmacologically in OA.

## Limitations

Current studies, despite the overall moderate to a high quality of evidence, still show some limitations. Most studies were conducted on animal models, not fully mimicking the complexity of the human microbiome. Notwithstanding the need for more human studies, all pieces of evidence here analysed have shown concordance of findings and show the same results of some clinical studies [5]. The heterogeneity of methods used to induce obesity and of the animal models is another limitation of the studies taken into account. However, all the validated models of obesity show the same results, and the same holds true for all the different animals used for *in vivo* studies. Our review is hoped to help direct future studies.

## Data Availability

The data that support the findings of this study are available from the corresponding author (GG), upon request.
